# Frontotemporal dementia and neurocysticercosis: A case report

**DOI:** 10.1590/S1980-57642012DN06010011

**Published:** 2012

**Authors:** Corina Satler, Elza Santos Maestro, Carlos Tomaz

**Affiliations:** 1 PhD, Laboratory of Neurosciences and Behavior, Institute of Biology, UnB, Brasília DF, Brazil.; 2 Psychologist, Geriatric Medical Center, University Hospital of Brasília, UnB, Brasília DF, Brazil.; 3 PhD, Full Professor, Laboratory of Neurosciences and Behavior, Institute of Biology, UnB, Brasília, Brasília DF, Brazil.

**Keywords:** progressive nonfluent aphasia, neurocysticercosis, neuropsychological assessment, frontotemporal lobar degeneration

## Abstract

We report a case of a 67-year-old woman with frontotemporal dementia (FTD) and a history of neurocysticercosis. After her retirement she showed progressive behavioral changes and neuropsychiatric symptoms with relative preservation of cognitive functioning. During the next three years, the patient manifested progressive deterioration of verbal communication gradually evolving to mutism, a hallmark of cases of progressive nonfluent aphasia.

## Abstract (pt)

Caso de uma mulher de 71 anos com demência frontotemporal e historia de neurocisticercose. Após sua aposentadoria ela apresentou progressivas mudanças comportamentais e sintomas neuropsiquiátricos, com relativa preservação do funcionamento cognitivo. Durante os seguintes três anos, a paciente foi desenvolvendo uma deteriorização progressiva da comunicação verbal evoluindo gradualmente a mutismo, marca que descreve um caso de afasia progressiva não fluente.

## INTRODUCTION

Frontotemporal dementia (FDT) is the most common form of primary degenerative dementia after Alzheimer's disease which affects individuals in middle age. This dementia occurs most commonly between the ages of 45 and 65 years^1^ and is associated with atrophy and neuronal loss affecting the frontal and temporal lobes of the brain.^2,3^

There are clinical subgroups of patients with frontotemporal lobar degeneration including a decline in behavior and executive functioning,^1^ semantic dementia,^4^ and progressive nonfluent aphasia.^5^ The three FTD syndrome variants are all characterized by the presence of behavioral and personality changes and/or aphasia with overlapping features.^6^

Frontotemporal dementia has an enormous impact on caregivers as it produces marked changes in personality, behavior, and communication abilities.^7^

Additionally, abnormal frontal lobe function is further reflected in patients' decreased insight and awareness of their disability or the consequences of their behavior.^8^

Neurocysticercosis (NCC) however is a parasitic infection of the human central nervous system (CNS) caused by the larval stage (Cysticercus cellulosae) of the pork tapeworm *Taenia solium,* which is associated to poor hygiene and basic sanitation conditions. From an epidemiological point of view, NCC can be found disseminated in different parts of the world, and has become an increasingly important emerging infection in the United States and developing countries of Latin America, Africa and Asia.^9^ Particularly in the developing world, NCC is the most common cause of acquired epilepsy.^10^

In Brazil, the greatest prevalence of NCC is found in the states of Paraná, São Paulo, Minas Gerais, Rio de Janeiro and Bahia.^11^

The clinical pleomorphism of NCC is mainly characterized by individual differences in the number and location of the lesions within the CNS and by variations in the severity of disease activity.^12^ NCC is a highly complex disease whose clinical manifestations and prognosis are related to various independent factors; the biological status of parasites (from live, active cysticerci to inactive granulomas and calcifications), the number and locations of lesions, and the degree of inflammatory response of the host to the parasites, which may range from immune tolerance to an intense inflammatory response.^13^ The severity of the disease can vary from an asymptomatic stage, discovered by incidental imaging studies, to a severe neurologic disorder.^14^

The process of degeneration of parasitic cysts involves a continuum that has been categorized into four histopathological stages: viable, colloidal, nodular-granular and calcified.^10^

The disease can cause many neurologic symptoms and present varied clinical and psychiatric manifestations. The clinical picture ranges from asymptomatic infections to severe life-threatening disease. Seizures are the most commonly reported symptom at presentation, occurring in up to 50-80% of NCC patients.^15^ Headaches secondary to intracranial hypertension and focal neurologic deficits are also common.^16^

NCC can present a wide range of neuropsychiatric symptoms, that can be isolated (e.g., mental confusion, hallucinations, *delirium*, depressive or anxious symptoms) or constitute a well-characterized disorder (e.g., major depression, minor depression, intermittent depression, mania, panic disorder, general anxiety disorder, phobias, personality disorder and dementia).^17,18^ Additionally, patients with NCC often display cognitive impairment. Mild to moderate cognitive dysfunction has been reported in up to 88% of NCC patients.^19^

Recently, Ciampi De Andrade et al., used a comprehensive neuropsychological battery to evaluate a group of patients with NCC and a group of matched healthy and epilepsy controls.^16^ The authors found impairment in multiple cognitive domains among patients with NCC prior to treatment compared with both control groups.

In this report, we discuss a case of a woman with FTD and progressive decline in her ability to use language who had a previous history of NCC. The longitudinal change in cognitive functioning is described along with behavioral and psychiatric features.

## CASE REPORT

A 67-year-old white woman, single, right-handed, with 12 years of schooling, gradually developed difficulties naming objects and understanding spoken language, accompanied by personality and behavioral changes. The only relevant familial antecedent was her mother who had dementia at 76 years old (her mother no longer recognized her), according to information from her relatives. There were no relevant familial psychiatric antecedents.

Her medical history revealed that in 1955, at the age of 11, she suffered from severe headaches, fainting and epilepsy and was diagnosed with NCC. According to her son (and main caregiver) there are no records of the kind of treatment or drugs used to treat the infection.

However, information recalled indicated that the patient initially had partial-onset seizures. Repeated seizures during the months that followed seemed to be associated with active cysts; that is, with the active phase of the disease.^9,20^ Additionally, reports suggest that calcified lesions are the most common clinical presentation of NCC associated with epileptic seizures^21^ and that seizures are the most frequent manifestation of cysts located in the brain parenchyma.^21,22^

**Figure 1:**
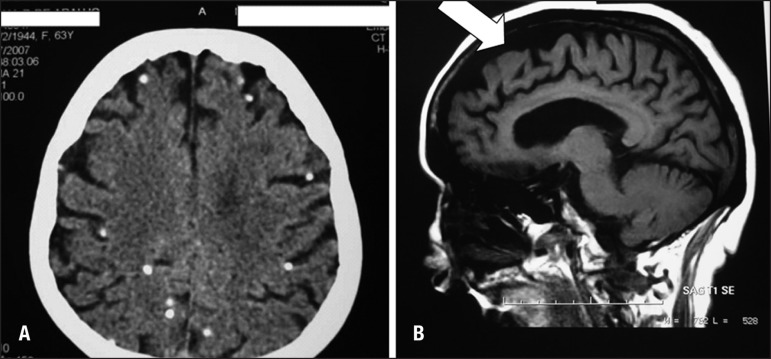
[A] Unenhanced computed tomography (CT Scan of the brain) showing multiple calciﬁed lesions. [B] Sagittal MR image, particularly the area near white arrow, shows the brain shrinkage common in FTD.

The patient presented radiographic signs of calcified NCC on CT Scan (Figure 1). The predominant finding was more than 20 calcified lesions located bilaterally in the brain cortex and sub-cortical regions.

It is important to note that the epileptogenesis in patients with neurocysticercosis can be attributed to several factors: inflammation, gliosis, genetics, and predilection for the cysts to travel to the frontal and temporal lobes.^23^

There is no evidence of an assessment of cognitive functioning indicating cognitive disturbances, although several studies have shown that a high percentage of patients present this deficit.^16,19^

During subsequent decades she led a normal life. However, in 2004 at the age of 60, after her retirement as a nursing technician, her son started to notice significant changes in her behavior.

The changes became particularly evident when the patient began going to the bank every day to reconfirm the balance of her checking account, arguing that someone could be stealing her money, and started presenting concurrent symptoms of persecutory ideas.

At the same time, he reported that she had exaggerated fears, anxiety and that she could not stay home alone. A religious awakening led her to spending hours praying and she argued with her son that she had to protect him from evil thoughts.

A geriatric assessment in 2005 revealed that the patient continued presenting excessive worry and tension. Behavioral control attempts were also reported and repetitive behaviors occurred in the verbal domain in the form of stereotyped use of words or phrases about religion. She was diagnosed with obsessive-compulsive disorder. Because the medication prescribed - Escitalopram, 20 mg daily caused side effects (nausea and stomach pain), Cloxazolam (2 mg daily) and Fluoxetina (20 mg daily) were prescribed.

In June 2006, a neuropsychological assessment was carried out to assess the cognitive functioning of the patient. Results indicated preservation of global cognition (Mini-Mental State Examination - MMSE), normal performance in executive and attention functions (Frontal Assessment Battery, and Clock Drawing Test - Part 1) and verbal fluency. In contrast, scores on a semantic memory task (Verbal Fluency Animals) and abstract reasoning (subtest Similarities) were just below the cut-off value (Table 1).

**Table 1:** Neuropsychological assessment.

Tests	June / 2006	February / 2009
CDR score	1	2
NPI total score	-	30
Instrumental ADL, n/8	-	26
IQCODE score, n/3.5	-	3.68
CDSD total score, n/8	-	10
GDS, n/5	3	No response
MMSE, n/30	30	17
Clock Drawing Test (part 1), n/10	8	7
Clock Drawing Test (part 2), n/10	-	8
Frontal Assessment Battery , n/18	14	-
Similarities (WAIS), n/10	5	-
Verbal Fluency FAS	28	-
Verbal Fluency Animals	9	-
Boston Naming Test, n/15	-	8
Token Test	-	Incomplete

ADL: Activities of Daily Living; CDR: Clinical Dementia Rating; CDSD: Cornell Depression Scale in Dementia; GDS: Geriatric Depression Scale; IQCODE: NPI: Neuropsychiatric Inventory.

At this time, she had a CDR of 0.5 and was taking Mirtazapine (30 mg daily).

An electroencephalogram (EEG) in 2007 indicated slowing of alpha activity and a presence of bursts of slow waves. These findings were associated with cortical and subcortical electrogenesis disorders. At the same time, a computed tomography scan of the head and a subsequent magnetic resonance imaging scan of the brain (2009) showed multiple intracerebral calcified lesions consistent with resolved NCC (Figure 1A) as well as brain volume reductions of all frontotemporal regions. respectively (Figure 1B).

Three years later, in February 2009, she attended the day-care hospital at the Geriatric Medical Center, Brasília University Hospital. An interdisciplinary team of social and health care professionals (a social worker, an occupational therapist, a nutritionist, a neuropsychologist and a geriatrician) examined the patient.

She was diagnosed with FTD and resolved NCC, based on clinical examination, cognitive and behavioral assessments, and neuroimaging studies.

At the time, her medication was aimed at treating gastroesophageal reflux disease with Pantoprazole (20 mg daily) and Domperidone (20 mg daily).

At the time of her admission, with CDR 2, she was grossly disorientated as to time and place. A neuropsychological evaluation was therefore conducted to investigate the progression of her disease. A written informed consent was obtained from her son in accordance with the ethical guidelines for research with human subjects (196/96 CNS/MS resolution).

During the interview, he stated that her behavior had gradually become more inadequate, presenting signs of disinhibition, loss of personal and social awareness (disregard for social norms such as touching unknown people when talking to them) and she waved to pictures on magazines. Her eating habits changed (overeating and addiction to sweets) and she presented fast progressive speech loss.

Regarding activities of daily living, upon application of the Informant Questionnaire on Cognitive Decline (IQCODE), he commented that she had significant impairment over the previous two years, mainly for those activities that allow independent life in a community.

Neuropsychiatric problems were identified including apathy, night-time behavior disturbances, and eating abnormalities - Neuropsychiatric Inventory.^24^

Although the scores obtained on the depression scale - Cornell Scale of Depression in Dementia,^25^ were above the cut-off value, the patient's medical history was not positive for depression.

Her ability to concentrate and stay focused when performing a task was found to be impaired during the neuropsychological testing. Moreover, she was disoriented in terms of place and time (MMSE), and presented difficulties with tasks that required organization, planning, symbolic and graphomotor representation, such as sequencing of numbers (Clock Drawing Test). She was not aware of her inability, as demonstrated by the absence of anxiety or frustration.

Visual tracking and gaze to verbal commands was without evidence of visual neglect or visual field cuts. Additionally, utilization behavior appeared over time (tendency to pick up and manipulate any object in the environment).

Specifically on the Boston Naming Test, it was evident that she had poor performance considering the cut-off value, indicating naming difficulties and suggesting neurogenic language deficits.^26^ Utilization behavior was again observed when she manifested the need to take the block of figures and draw each figure with her finger several times.

A significant impairment in cognitive functioning in relation to premorbid abilities was noted. Although memory difficulty was evident, neither verbal nor visual memory could be tested in depth because of the communication problems evident.

She showed difficulties processing complex instructions and following logic or complex sentences, as well as with production of speech, having specific difficulty comprehending and repeating, and also showed long response latency. She was able to produce only single words, but displayed no ability to produce sentences or even brief meaningful phrases. Overall, her son confirmed these changes, citing that her ability to use language had been declining over the last 2 years.

Regarding motor functions, she was able to initiate a motor response to verbal instructions, correctly executing simple commands (Token test). She responded consistently to one-step commands and could perform the task when given a concrete visual cue, more specifically, the fist-edge-palm test (Luria's 3-steps motor program), for short periods of time. However, it was observed that she was easily distracted and it was necessary to repeat the instructions on some occasions. She presented difficulty responding to two-and three-step commands and often became perseverant in her responses. She could not correctly reproduce complex sequences of hand movements when presented by the examiner, repeating whole sets of previously executed behaviors suggesting loss of inhibitory control over behavior and sustained attention failure (Table 1).

**Follow-up**. A further medical consultation was scheduled in August 2009, but the patient did not attend. The next contact was possible only in February 2010. On this occasion, her son explained to us that the patient required home care and that a nursing home was facilitating self-care activities.

During the a home visit assessment, the physician evaluated the patient and highlighted that her language impairment had developed to mutism with explosive shrieks and guttural sounds; reported additionally that she was unable to communicate or demonstrate any capacity for comprehension and that she had controlled feeding owing to difficulties swallowing and increased hyperorality.

Further imaging studies were not feasible.

## DISCUSSION

We report and illustrate a case of NCC in which a patient gradually evolved to FDT and mutism over several years.

NCC represents one of the most serious public health problems in developing nations. Many studies have described a continuum of cognitive decline in NCC, ranging from cognitive impairment on at least one test (without functional impairment) extending to dementia.^16,18,19,27^ Although it is unclear how cysticercotic NCC lesions exert their effects on cognition, at least some of the mental status changes described in clinical reports could be explained by partial seizures, the mass effect of cysts, and increased intracranial pressure.^28^

It is important to point out that although cognitive dysfunction is relatively common in NCC, cognitive and quality of life deficits improve with time.^28^

Specifically in our case, we may assume the absence of a sufficiently severe cognitive impairment to significantly interfere with activities of daily living considering that the patient had a good educational and work history. However, the possibility of cognitive sequelae associated with the NCC cannot be ruled out.

Thus, NCC can be identified as a comorbid condition associated with FTD excluding any influence of this disease on the possible development of FTD.

At the age of 60 years, after her retirement, the behavioral changes and personality alterations only became evident with intact and only mildly impaired cognitive functions.

However, the early onset of FTD was misdiagnosed because the symptoms (particularly behavioral changes) were associated with obsessive-compulsive disorder. Studies have reported that FTD in early stages can mimic other disorders leading to consequent misdiagnosis.^29^

Shortly after, she began gradually showing a predominant frontal lobe symptom pattern with accentuated behavioral symptoms e.g., repetitive and stereotyped behaviors and utilization behavior; dietary changes (overeating), and decline in personal hygiene and grooming;^3,29,30^ personality changes, loss of social awareness and sense of what is proper, and a total loss of insight.^8^ Additionally, she presented progressive nonfluent aphasia, that evolved to mutism.^29,30^

In this case, akin to most other reported cases of primary progressive aphasia, the language disorder progressed to dementia with involvement of multiple cognitive domains, specifically within the spectrum of frontotemporal dementia pathology.^3^

In summary, it is important to note that, according to detailed history taken from family members and medical reports highlighting early decline in behavior and personality changes, the clinical profile of the case seemed to be more closely associated with the behavioral variant of FTD. However, over a three-year period progressive language deterioration occurred with loss of generative capacity evolving to mutism, a characteristic of progressive nonfluent aphasia.
